# Supplementary material for: New insights into the lifestyle of Allosaurus (Dinosauria: Theropoda) based on another specimen with multiple pathologies
Source: PeerJ. 2015 May 12;3:e940. doi: 10.7717/peerj.940 (PMC4435507; doi:10.7717/peerj.940)
Supplement: Supplemental Information 1 — This file contains figures from the main text without interpretive lines of pathologies for comparison. [file peerj-03-940-s001.pdf]

Supplementary information

New insights into the lifestyle of *Allosaurus* (Dinosauria: Theropoda) based on another specimen with multiple pathologies

Christian Foth[1,2,3] , Serjoscha W. Evers[3,4], Ben Pabst[5], Octávio Mateus[6,7], Alexander Flisch[8], Mike Patthey[9], Oliver W. M. Rauhut[1,2]

[1] SNBS, Bayerische Staatssammlung für Paläontologie und Geologie, Richard Wagner-Str. 10, D-80333 München

[2] Department of Earth and Environmental Sciences, Ludwig-Maximilians-Universität, Richard-Wagner-Str. 10, D-80333 München, Germany

[3] Department of Geosciences, University of Fribourg/Freiburg, Chemin du Musée 6, 1700 Fribourg, Switzerland

[4] Department of Earth Sciences, University of Oxford, South Parks Road, Oxford OX1 3AN, UK

[5] Sauriermuseum Aathal, Zürichstr. 69, CH-8607 Aathal-Seegräben, Switzerland

[6] CICEGe, Faculdade de Ciências e Tecnologia, FCT, Universidade Nova de Lisboa, 2829-516 Caparica, Portugal

[7] Museu da Lourinhã, Rua João Luis de Moura, 2530-157 Lourinhã, Portugal

[8] Swiss Federal Laboratories for Materials Science and Technology, Center for X-ray Analytics, Überlandstrasse 129, CH-8600 Dübendorf, Switzerland

[9] Vetsuisse Fakulty, Universität Zürich, Winterthurerstrasse 260, CH-8057 Zürich, Switzerland

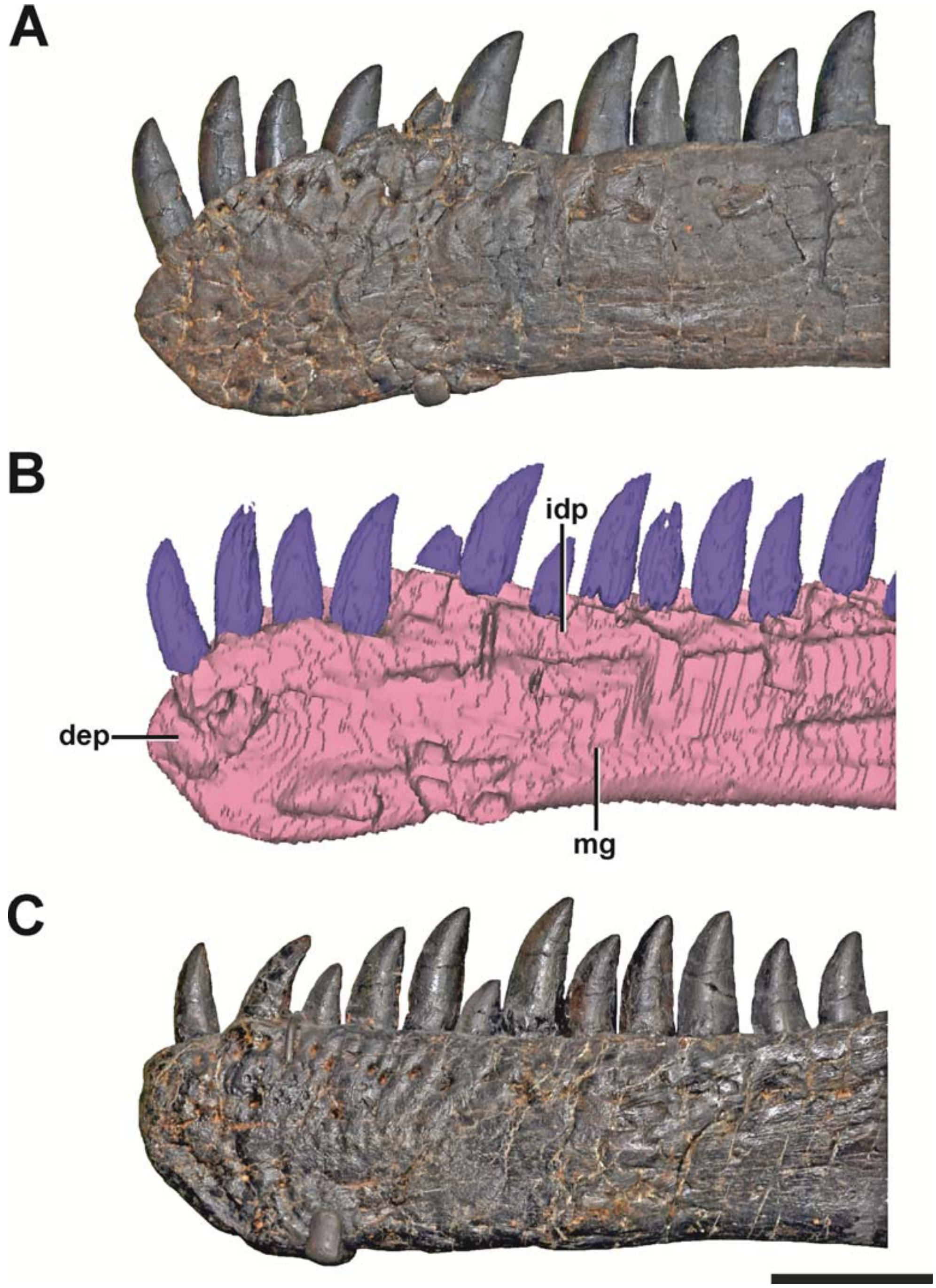

Figure S1 The dentaries of SMA 0005. (A) Left dentary with pathologic anterior end in lateral view. (B) Medial side of the left dentary with pathologic anterior end in mirrored view. (C) Right dentary in mirrored view, showing the normal condition for *Allosaurus*. Note that most teeth in both dentaries are not original, but glued to the internal margin of the dentaries. **Abbreviations: dep** depression, **idp** interdental plates, **mg** Meckelian groove. Scale bar = 5 cm.

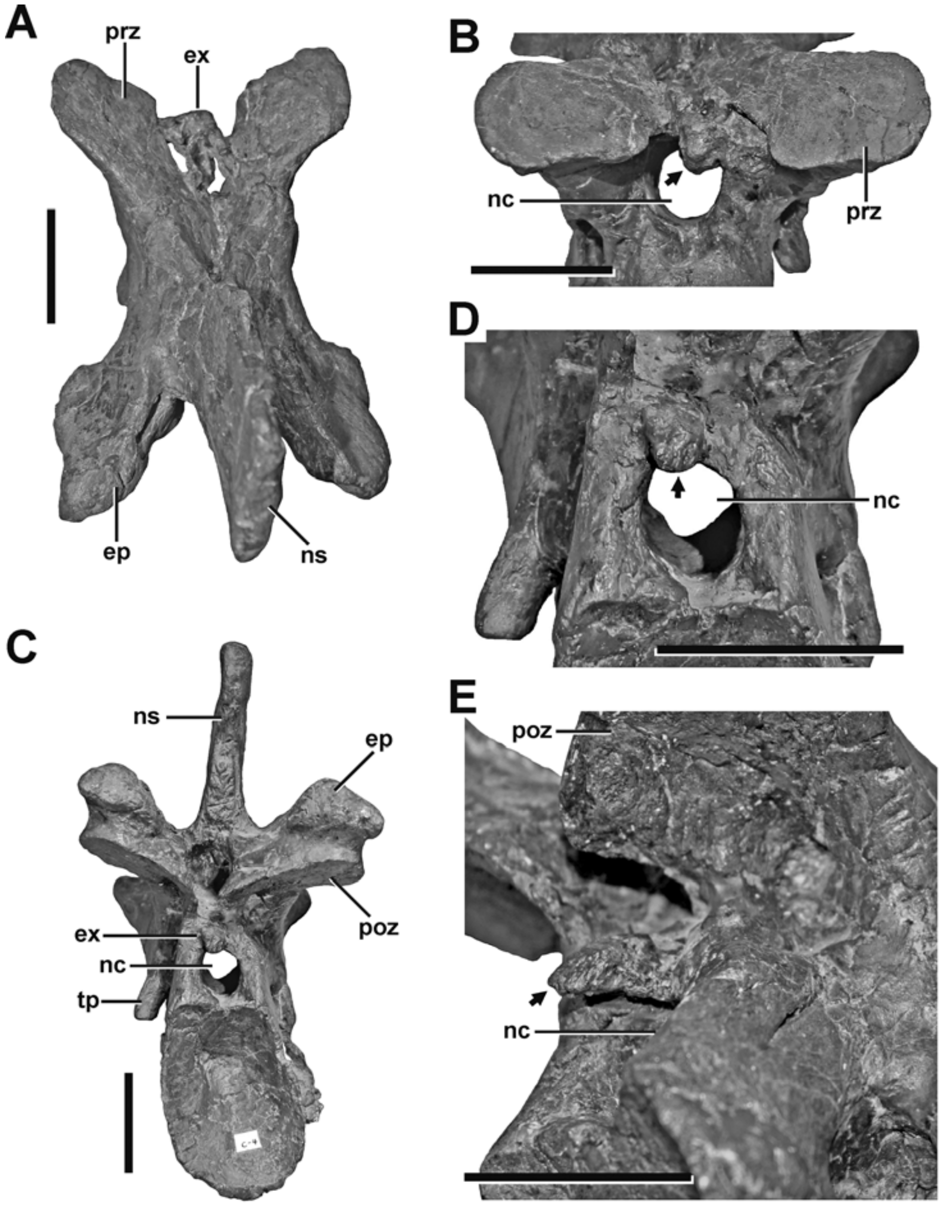

Figure S2 Fourth cervical of SMA 0005. (A) Fourth cervical in dorsal view, showing a pathologic exostosis (possible osteochondroma) between the prezygapophyses. (B) Possible osteochondroma from anterior view marked by a dotted line. (C) Fourth cervical in posterior view, showing another exostosis (possible inflammatory ossification) above the neural canel (arrow). (D) Possible inflammatory ossification in close-view (arrow). (E) Possible inflammatory ossification in posterolateral view (arrow). **Abbreviations: ep** epipophysis, **ex** exostosis, **nc** neural canal, **ns** neural spine, **poz** postzygapophysis, **prz** prezygapophysis, **tp** transverse process. Scale bar = 5 cm.

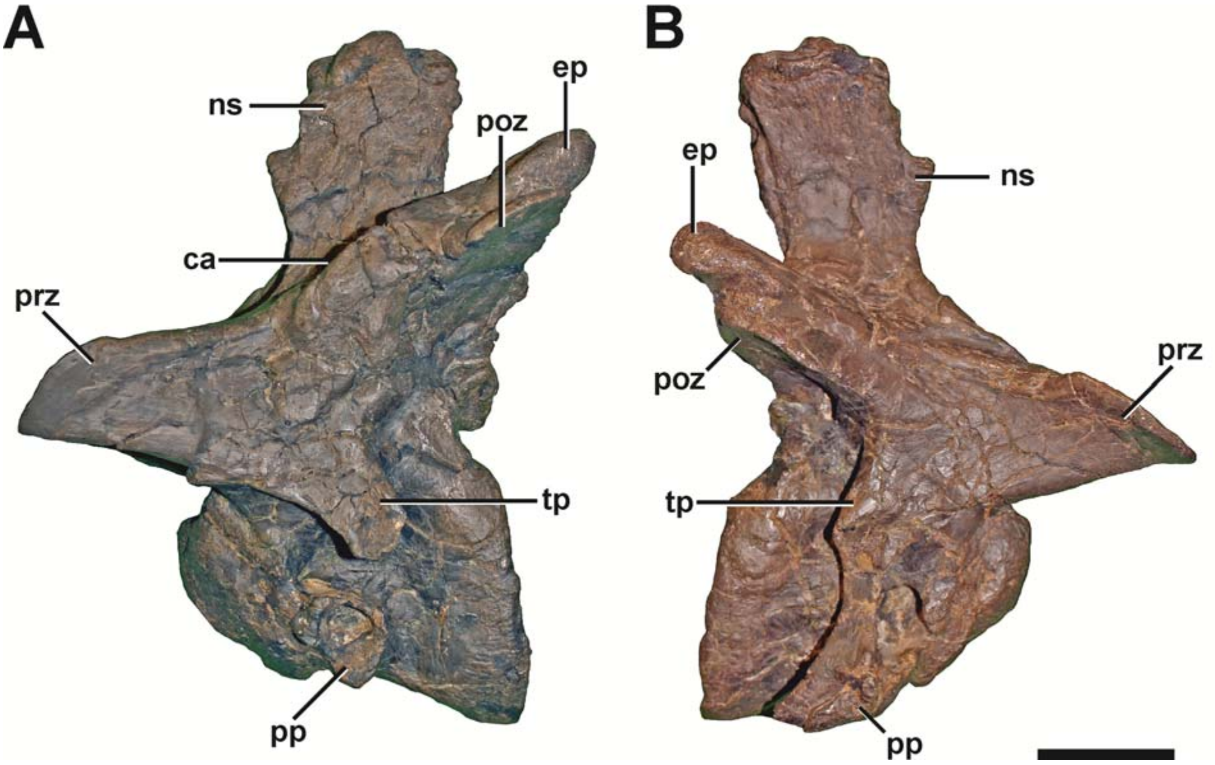

Figure S3 Fifth cervical of SMA 0005. (A) Fifth cervical in left lateral view, showing the pathologic postzygapophysis. (B) Fifth cervical in right lateral view, showing the normal condition of the postzygapophysis. **Abbreviations: ca** callus, **ep** epipophysis, **ns** neural spine, **poz** postzygapophysis, **pp** parapophysis, **prz** prezygapophysis, **tp** transverse process. Scale bar = 5 cm.

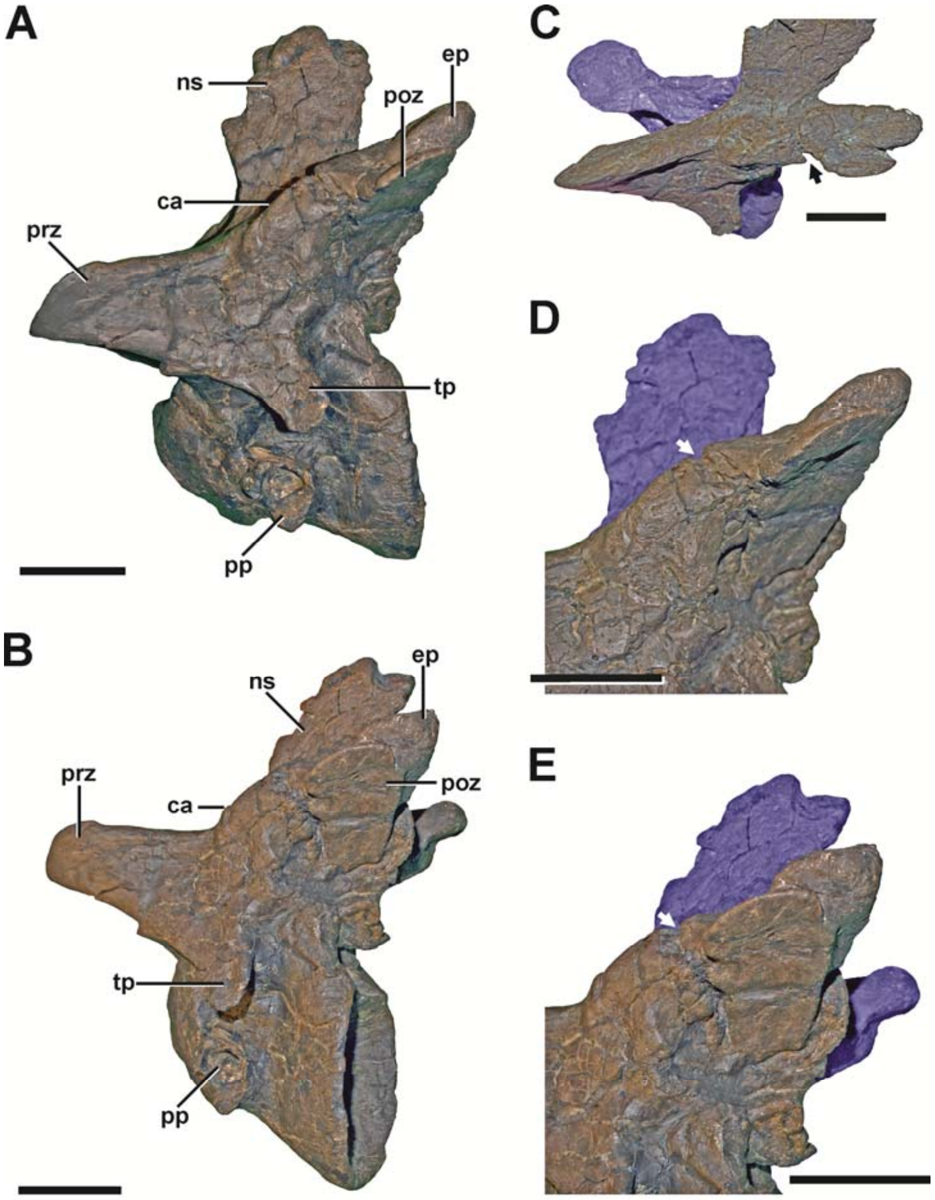

Figure S4 Fifth cervical of SMA 0005. (A) Fifth cervical in left lateral view, showing the callus and the fracture at the base of the left postzygapophysis. (B) Fifth cervical in lateral view in posterolateral view. (C) Callus and fracture (arrow) in laterodorsal view. (D) Callus and fracture (arrow) in lateroventral view. (E) Callus and fracture (arrow) in oblique posterolateral view. Structures of minor interest in the neural arch are colored transparently for cover. **Abbreviations: ca** callus, **ep** epipophysis, **ns** neural spine, **poz** postzygapophysis, **pp** parapophysis, **prz** prezygapophysis, **tp** transverse process. Scale bar = 5 cm.

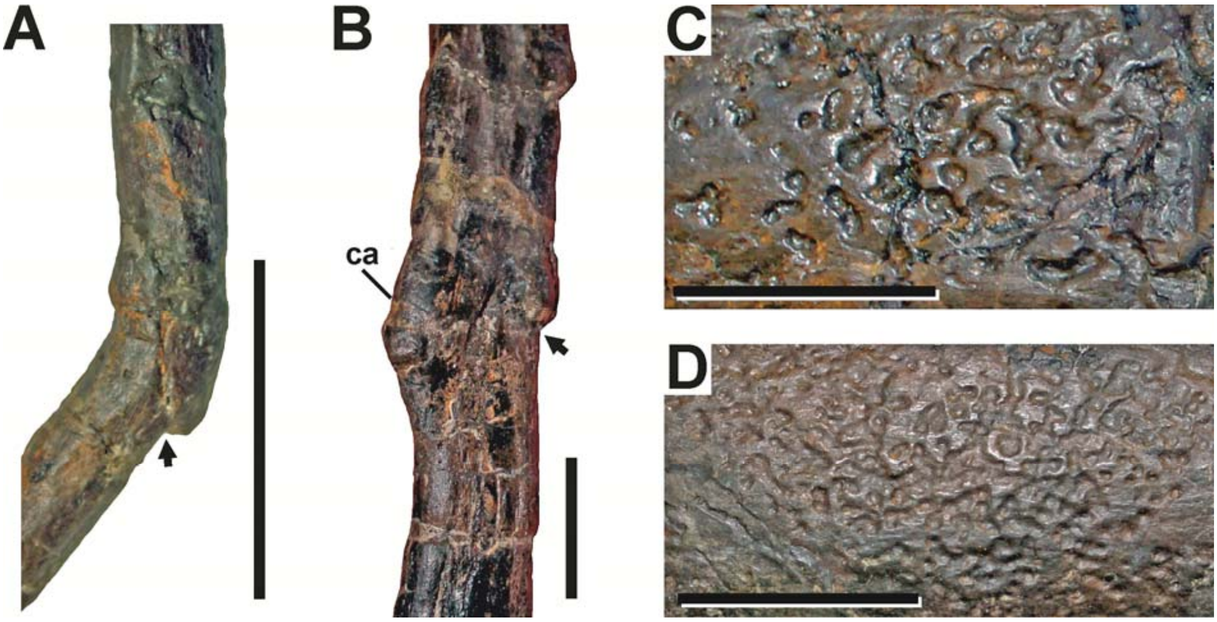

Figure S5 Pathologic ribs and cortical traces in SMA 0005. (A) Fourth cervical rib from the right side with fracture (arrow). (B) Seventh dorsal rib from the left side with fracture (arrow). (C) Cortical traces in the right ischium. (D) Cortical traces in the left scapula. **Abbreviations: ca** callus. Scale bar = 2 cm.

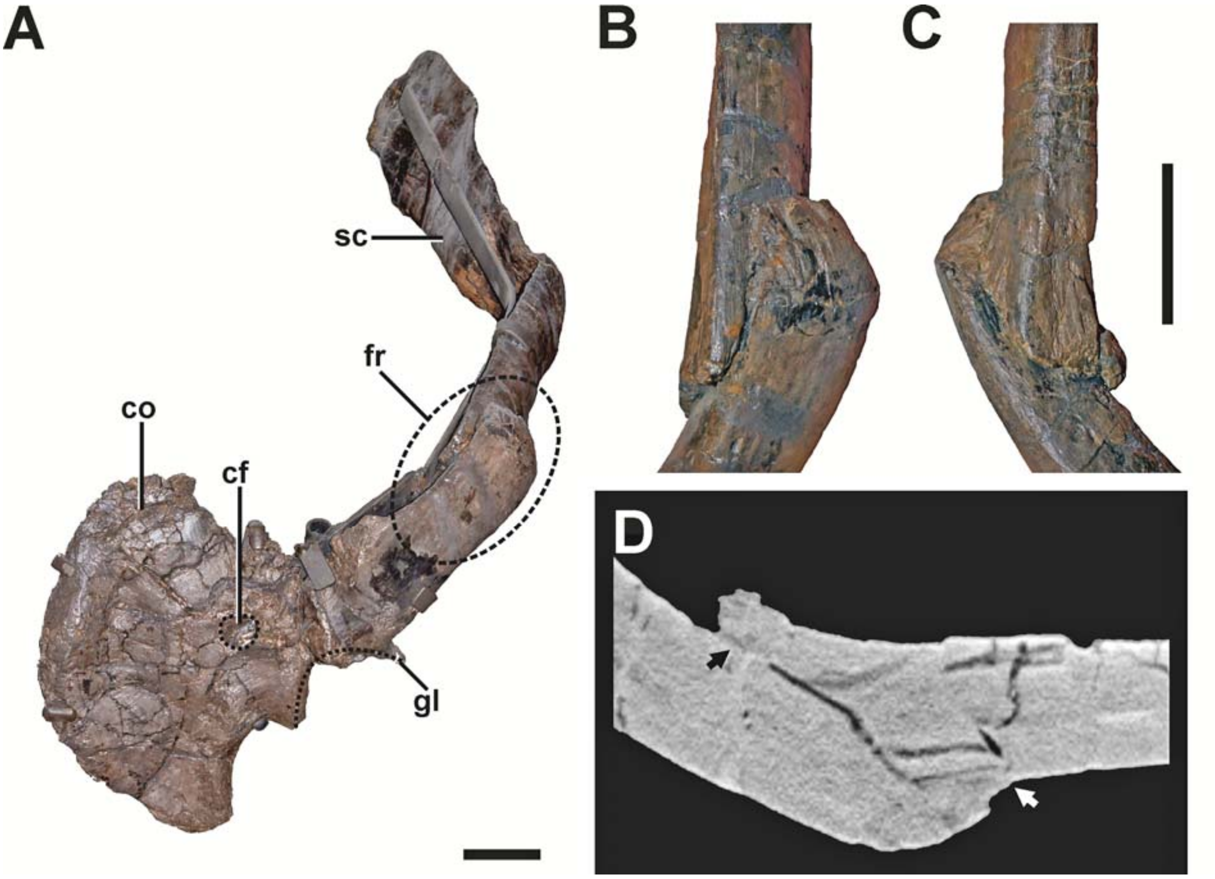

Figure S6 Pathologic scapula of SMA 0005. (A) Left scapula in anterolateral view, showing the fractured area of the scapula blade (dashed line). (B) Fracture in dorsal view. (C) Fracture in ventral view. (D) Tomogram of the scapula, showing that only the fracture ends constitute a fused bridge between the fracture elements (arrows), which is consistent with a pseudarthrosis. **Abbreviations: co** coracoid, **cf** coracoid foramen, **fr** fracture, **gl** glenoid facet, **sc** scapula blade. Scale bar = 5 cm.

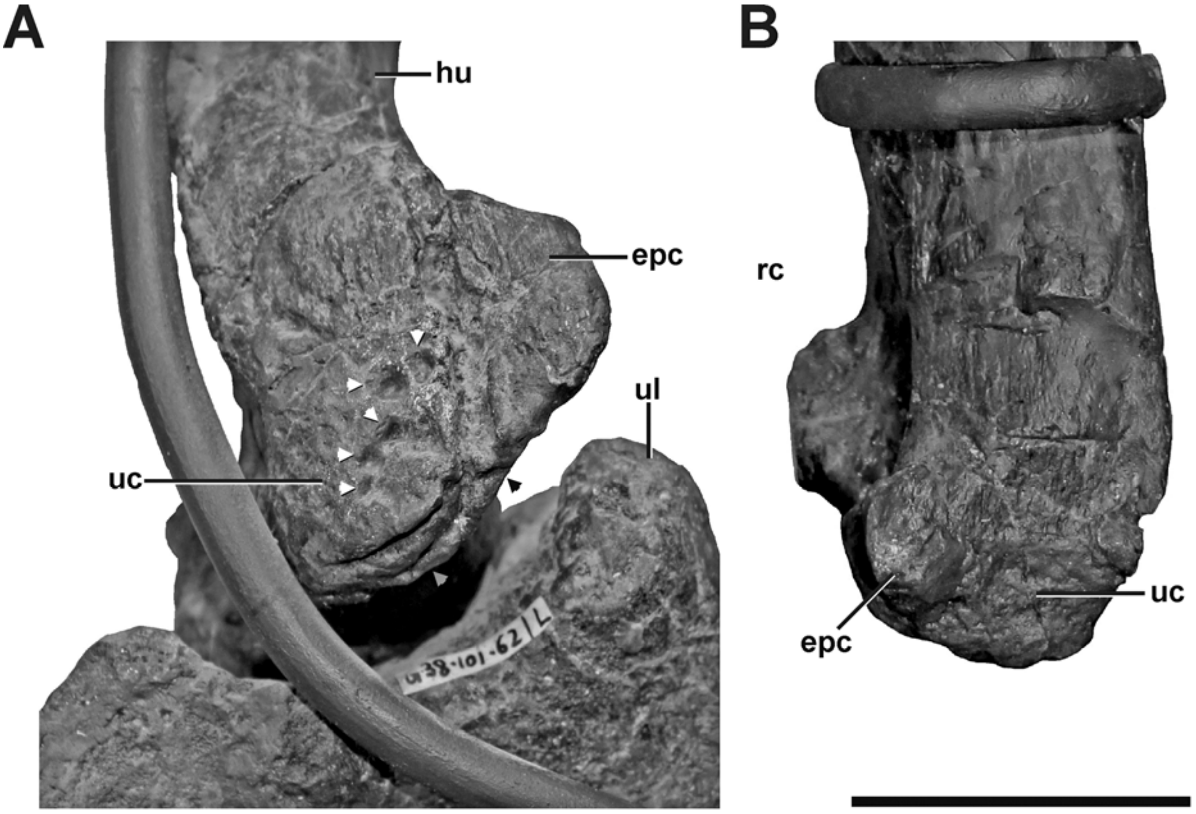

Figure S7 Left and right humerus of SMA 0005. (A) Distal portion of the left humerus in anteromedial view, showing idiopathic pathologies. The pathologies contain an irregular cortical texture with numerous depressions (white arrows), a deep oblique groove toward the anterior aspect of the medial side (black arrow), and two sharp, trough-like marks on the ventral surface of the ulnar condyle (grey arrow). (B) Distal portion of the right humerus in anterior view. **Abbreviations: epc** epicondyle, **hu** humerus, **ul** ulna, **uc** ulnar condyle. Scale bar = 5 cm.

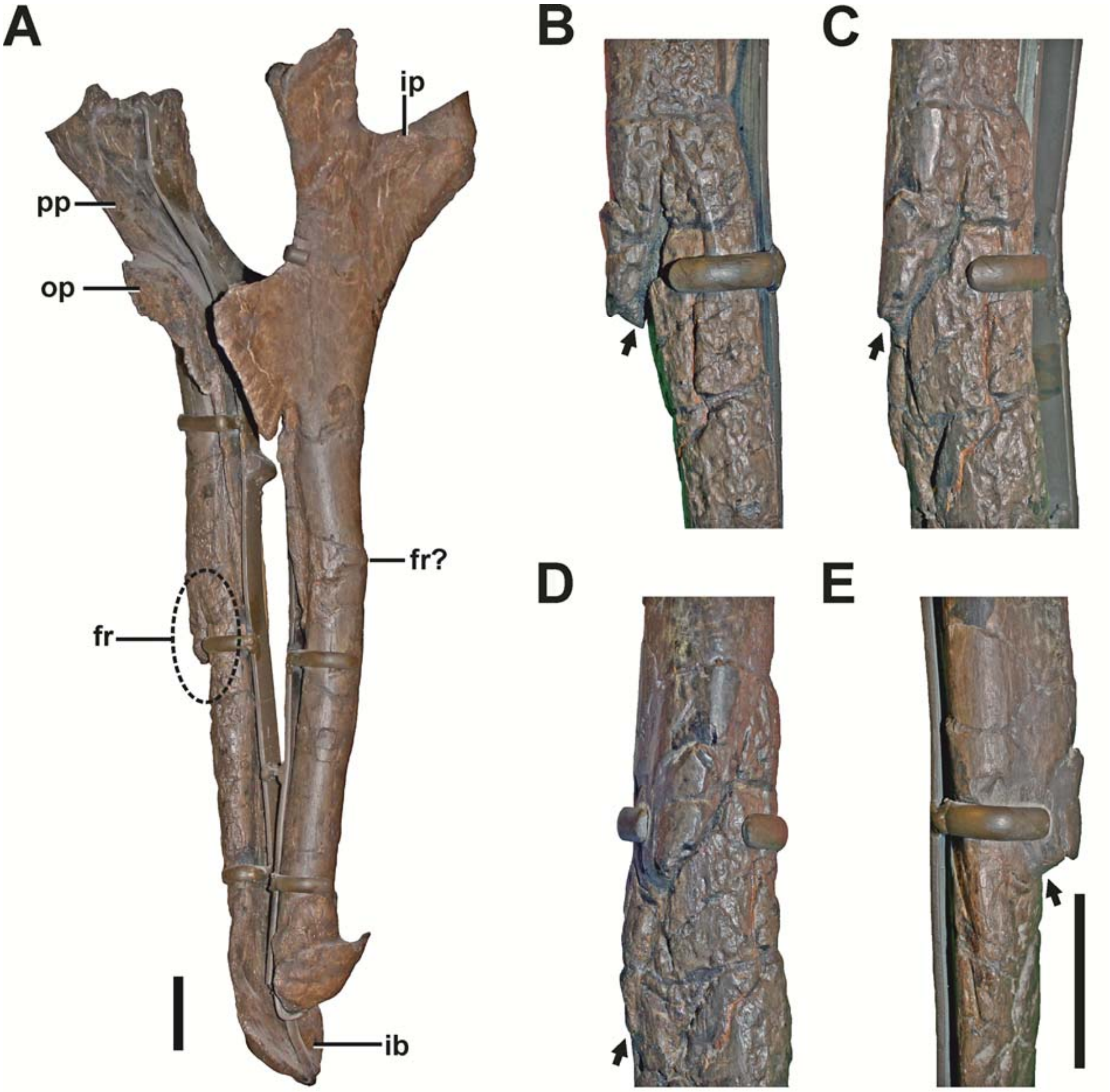

Figure S8 Ischium of SMA 0005. (A) Both Ischia in anterolateral view, showing the oblique fracture at the shaft of the right ischium. (B) Interfragmentary gap (arrow) of the right ischium in anterior view. (C) Fracture (arrow) of the right ischium in anterolateral view. (D) Fracture (arrow) of the right ischium in lateral view. (E) Fracture (arrow) of the right ischium in posterior view. **Abbreviations: fr** fracture, **ib** ischial boot, **ip** ischial peduncle, **op** obturator process **pp** pubic peduncle. Scale bar = 5 cm.

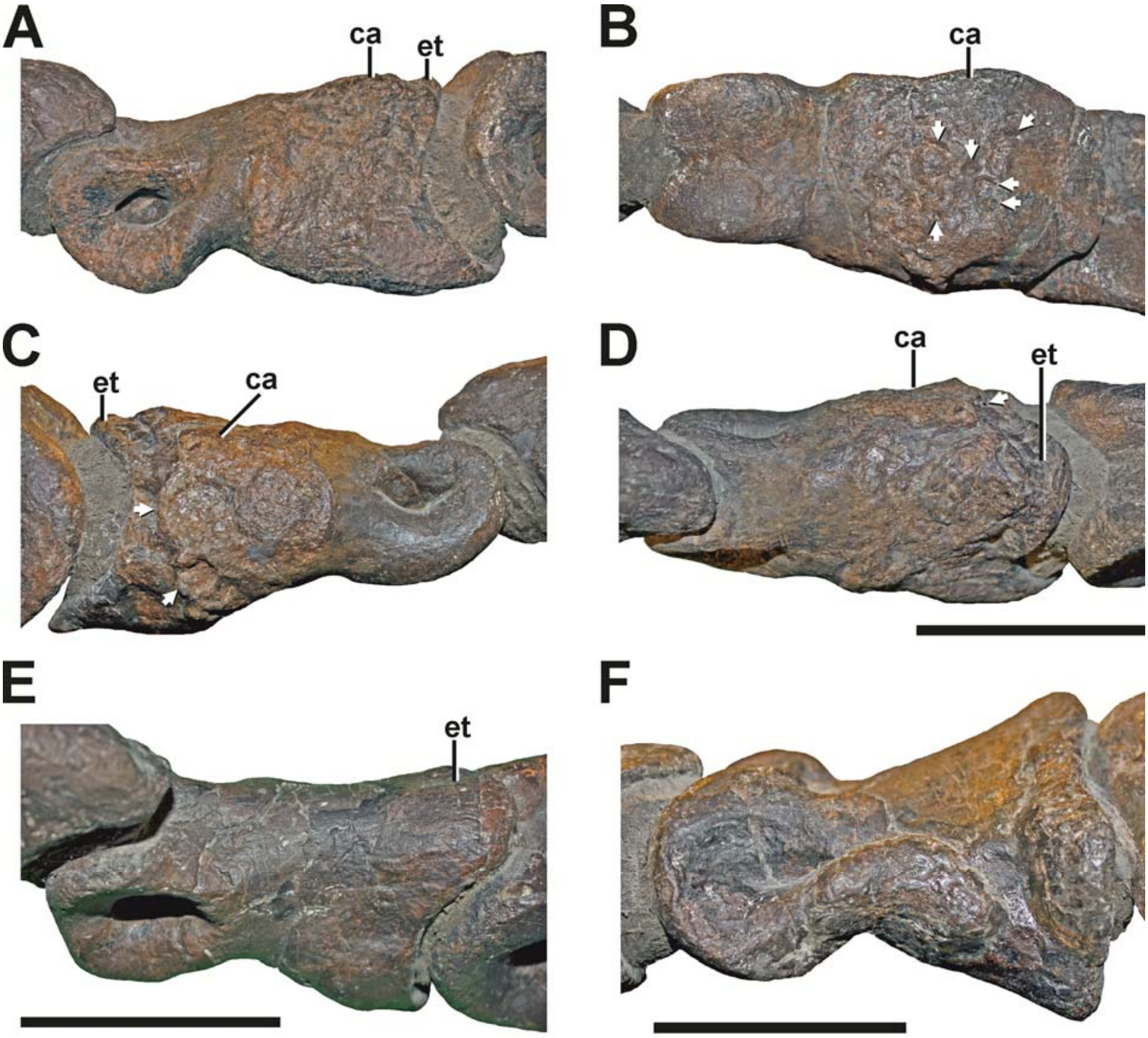

Figure S9 Pathologic phalanges in SMA 0005. (A) Left pedal phalanx II-2 from lateral view, showing the callus and the reduced extensor tubercle. (B) Left pedal phalanx II-2 from ventral view, showing the callus and multiple small depressions (arrows). (C) Left pedal phalanx II-2 from medial view, showing the callus and two large depressions (arrows), possibly indicating a secondary infection of the bone. (D) Left pedal phalanx II-2 from dorsal view, showing the callus and one of the two large depressions on the medial side. (E) Right pedal phalanx II-2 from mediodorsal view, showing the normal condition and size of the extensor tubercle. (F) Left pedal phalanx IV-1 in lateral view, showing two idiopathic bulbous swellings. **Abbreviations: ca** callus, **et** extensor tubercle. Scale bar = 5 cm.
